# Electrocatalytic Methods for Ammonia Production: A New Open Special Issue in *Materials*

**DOI:** 10.3390/ma15196609

**Published:** 2022-09-23

**Authors:** Zhefei Pan

**Affiliations:** Department of Mechanical Engineering, The Hong Kong Polytechnic University, Kowloon, Hong Kong, China; zhefei.pan@polyu.edu.hk

“*Electrocatalytic Methods for Ammonia Production*” is a new open Special Issue in *Materials*, which aims to publish original research papers, perspectives and review articles on theoretical and applied research, and shed an inspiring light on electrocatalytic methods of ammonia production.

Ammonia is an important chemical in agriculture, pharmaceutical and textile industries, which is industrially produced via a Haber–Bosch process that converts atmospheric nitrogen to ammonia by a reaction with hydrogen using a metal catalyst under high temperatures and pressures [1]. This significant achievement was awarded three Nobel prizes in chemistry in 1918, 1931 and 2007. However, the Haber–Bosch process annually consumes more than 1% of the global fossil fuels to create the necessary reaction conditions of high temperatures and pressures and release carbon dioxide into the atmosphere [2].

In response to these energy and environmental consequences, much attention has been paid to developing green and sustainable ambient ammonia synthesis technologies. Electrocatalytic nitrogen reduction is a promising alternative approach, which is capable of producing ammonia at a large scale under ambient conditions. Since nitrogen is a stubbornly stable molecule, breaking it up typically requires an efficient electrocatalyst to achieve high-performance ammonia production technology. Therefore, it is critically important to develop an efficient and cost-effective electrocatalyst for electrochemical nitrogen reduction.

The research interest of this Special Issue, “*Electrocatalytic Methods for Ammonia Production*” includes, but is not limited to, the following: fundamental aspects on mathematical modeling and simulation, newly developed characterization technologies, novel catalyst synthesis and modification, and innovative reactor and system designs.
